# Effectiveness and Usability of a Web-Based Mindfulness Intervention for Families Living with Mental Illness

**DOI:** 10.1007/s12671-016-0653-2

**Published:** 2016-12-08

**Authors:** Sigrid Stjernswärd, Lars Hansson

**Affiliations:** Department of Health Sciences, Lund University, Box 157, 221 50 Lund, Sweden

**Keywords:** Burden, Caregivers, Mindfulness, Self-compassion, Stress, Usability

## Abstract

Families living with mental illness express needs of support and experiences of burden that may affect their own health detrimentally and hence also their ability to support the patient. Mindfulness-based interventions have shown beneficial health effects in both clinical and healthy populations. The aim of the current study was to explore the effectiveness and usability of a web-based mindfulness program for families living with mental illness, which was first tested in a feasibility study. The study was designed as a randomized controlled trial with an experiment group and a wait-list control group with assessments on primary and secondary outcomes at baseline, post-intervention, and at a 3-month follow-up. Significant positive improvements in mindfulness and self-compassion, and significant decreases in perceived stress and in certain dimensions of caregiver burden were found, with good program usability. Easily accessible mindfulness-based interventions may be useful in addressing caregivers’ needs of support and in preventing further ill health in caregivers. Further studies are needed, among others, to further customize interventions and to investigate the cost-effectiveness of such programs.

## Introduction

Mental disorders represent an increasing burden for health care and society, with an estimated incidence in the EU population ranging from 27 to 38.2% (Wittchen and Jacobi 2005; Wittchen et al. 2011) and detrimental effects on health and well-being for both patients and their families. Families can act as facilitators to recovery when they offer support and motivation, but also as barriers and stressors when they display stigma and lack of understanding (Aldersey and Whitley 2015). Caregivers report positive experiences of caregiving, such as friendship, personal development, and a sense of achievement (Cormac and Tihanyi 2006). However, their situation can be marked by high levels of distress, among others, in relation to the patient’s behavior, care duties, own fears, and worries (Cormac and Tihanyi 2006). Caregivers report poorer mental health and more psychiatric symptoms than non-caregivers (Smith et al. 2014a). The need for vigilance, a sense of unpredictability and uncontrollability, but also secondary stress in relationships and professional life contribute to adversely affect caregivers’ health (Phillips et al. 2009). Caregivers can find themselves adjusting their life to the patient’s needs and overlooking own needs, with negative effects on health and quality of life (Skundberg‐Kletthagen et al. 2013; Stjernswärd and Östman 2008). Relating to someone with a mental illness (MI) can be a source of stressful interactions, and taking responsibility for automatic reactions and one’s own part in relationships that can enhance interpersonal stress is essential (Kabat-Zinn 2009). Effective coping strategies may improve families’ ability to cope with the stresses associated with MI and caregiving in daily life (Skundberg‐Kletthagen et al. 2013). If the patient feels good, so does the family, and vice versa (Wright and Leahey 2012). Early identification of caregivers to tailor information and support strategies is essential (Smith et al. 2014a) to prevent further ill health. One of these support strategies can be mindfulness-based strategies.

Mindfulness refers to “the awareness that emerges through paying attention on purpose, in the present moment, and non-judgmentally to the unfolding of experience moment by moment” (Kabat‐Zinn 2003, p.145). Mindfulness-based interventions (MBI), whether face-to-face (de Vibe et al. 2012) or online (Boettcher et al. 2014; Stjernswärd and Hansson 2016a, b), have shown beneficial health outcomes for both clinical (Boettcher et al. 2014; Chadwick et al. 2005) and non-clinical populations (Glück and Maercker 2011; Krusche et al. 2012). MBI show positive effects in terms of mindfulness, personal development (e.g., empathy, coping), quality of life, somatic health outcomes (de Vibe et al. 2012), and in the prevention of depression relapses (Kuyken et al. 2016). Further beneficial effects include decreased levels of stress (Glück and Maercker 2011; Krusche et al. 2012), anxiety (Goyal et al. 2014), and depression (Boettcher et al. 2014; Goyal et al. 2014), and positive psychological effects such as increased well-being and behavioral regulation, and decreased emotional reactivity (Keng et al. 2011). Higher levels of mindfulness also seem to be associated with higher relationship satisfaction and an enhanced ability to respond constructively to relationship stress, including better communication capacity (Barnes et al. 2007). Although findings are encouraging, research is at early stages with methodological flaws (e.g., lack of control group and standardized interventions) and more research of high quality is needed to draw conclusions about effectiveness (Fish et al. 2016). MBI have shown positive effects for caregivers of the frail elderly and of persons with a chronic condition in terms of decreased levels of anxiety and/or depression (Hou et al. 2013; Paller et al. 2015), stress, and caregiver burden (Epstein-Lubow et al. 2011; Pagnini et al. 2015; Stjernswärd and Hansson 2016a). Even briefer MBI appear to have beneficial effects (Boettcher et al. 2014; Carmody et al. 2009; Stjernswärd and Hansson 2016a, b; Vesa et al. 2016; Zeidan et al. 2010).

The cultivation of self-compassion, towards oneself and others, is more or less explicit and focused upon in different types of meditation practices (Hölzel et al. 2011). In mindfulness-based stress reduction programs (MBSR), associated with increases in self-compassion, the latter is implicitly and explicitly interwoven into meditation instructions (e.g., bring back attention with gentleness, explore what feels good, take care of yourself when encountering suffering, etc.) (Hölzel et al. 2011). Self-compassion has three components: (1) self-kindness versus self-judgment, (2) common humanity versus isolation, and (3) mindfulness versus overidentification or avoidance (Neff 2003). Compassion meditations contribute to acceptance and a non-judgmental and caring attitude towards the self and others (Kabat-Zinn 2009) and may hence be useful for caregivers that need to tackle inter-relational stress, with even relatively short training time resulting in positive effects of psychological functioning (Hofmann et al. 2011). Brevity may help prevent additional stressful time-commitment, which may be a reason for intervention drop-out (Shapiro et al. 2005).

Limited resources, exhaustion, transportation, and stigma are common barriers to help-seeking and treatment. Customized and user-friendly web-based interventions that are easily available at the convenience of caregivers may help overcome such barriers. The extent to which a specific user can use a specific product to reach specific goals, with purposefulness, effectiveness, and satisfaction, in a given context refers to its usability (International Organization for Standardization 1998). A web-based mindfulness intervention was tested in a prior feasibility study, with promising results in terms of increased levels of mindfulness and self-compassion, and reduced levels of caregiver burden and perceived stress (Stjernswärd and Hansson 2016a). The intervention showed good acceptance, feasibility, usability, and user value, with ease of use and convenience of access representing strong motivators for use (Stjernswärd and Hansson 2016b). The aim of the current study was to assess the effectiveness and usability of the mentioned web-based mindfulness intervention in a randomized controlled trial including families of a person with MI, with mindfulness as the primary outcome and caregiver burden, perceived stress, and self-compassion as secondary outcomes.

## Method

### Participants

**Fig. 1:**
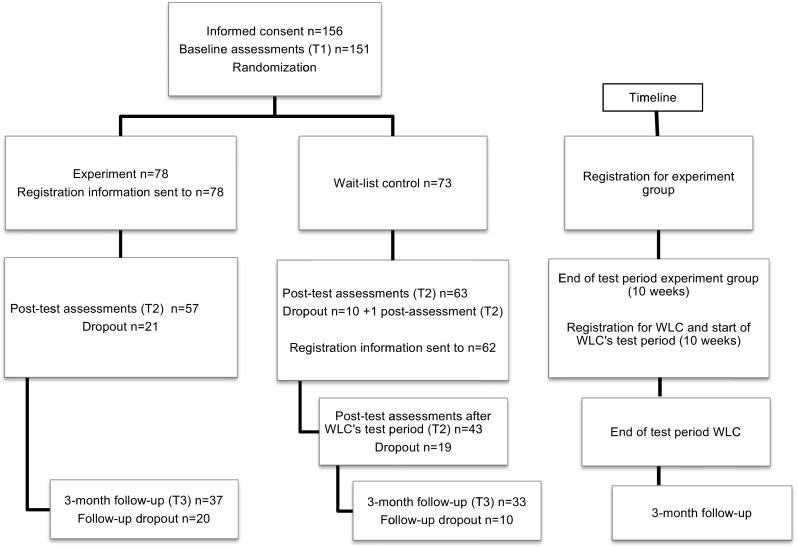
Total sample and dropout

Participants were recruited through advertisement in papers, newsletters, online, social media, and clinics/organizations with interests in caregivers. Information about the study and informed consent were available online. The inclusion criteria were age >18, being a relative/significant other to a person with MI (mixed diagnosis/as reported by participants), having access to a computer/Internet, and to understand Swedish. The exclusion criteria were having prior experience of mindfulness meditation and an own severe MI that requires other professional treatment. Participants who fulfilled the inclusion criteria and submitted an informed consent were randomized (randomizer.org) into an experimental group and a WLC after answering the baseline assessments (T1) (Fig. 1). A power calculation based on the primary outcome measure (mindfulness) from the feasibility study showed that100 participants (50 per arm) would be an adequate sample size to identify a medium effect size using a 80% probability and *p* <0.05 as a marker of significant differences. We intended to include 140 persons, accounting for a drop-out rate of around 30%.

Out of the 156 participants who signed an informed consent and fulfilled the inclusion criteria, 151 answered the sociodemographic and baseline questionnaires (T1) and were randomized into an experiment group and a WLC with respectively 78 and 73 participants. Thereafter, participants in the experiment group were sent an e-mail with instructions for registration to the web-based mindfulness program. Participants in the WLC were sent similar instructions once the post-test questionnaires following the first test period (T2) were answered, which also represented their baseline assessments prior to their own test period. The latter followed immediately after the named assessments at T2. There were no significant sociodemographic differences between the groups. The majority of participants were women, aged 40–69 (mean = 54), and a parent to the patient (56 and 46% in the respective groups), but also partners (19%/18%), adult children (10%/24–25%), siblings (10%/6%), and persons with another relationship to the patient (4%/1%) were represented (Table 1).

**Table 1:** Background characteristics of the baseline sample (*n* = 151)

Baseline *n* = 151	Experiment group *n* = 78		WLC *n* = 73	
	*n*	%	*n*	%
Men/women	8/70	10/90	10/63	14/86
Age				
20–29 30–39 40–49 50–59 60–69 ≥70 Missing Married or in a relationship/single	5 4 20 25 18 6 – 56/22	6 5 26 32 23 8 – 72/28	2 5 13 25 18 9 1 49/24	3 7 18 34 25 12 1 63/33
Relationship to the patient				
Father/mother Son/daughter Brother/sister Partner (male/female) Other relationship	44 8 8 15 3	56 10 10 19 4	36 18 5 13 1	49 25 7 18 1
Shared household with the affected person				
Yes No Sometimes	36 27 15	46 35 19	26 30 17	36 41 23
Living situation				
In a city/town On the country-side	69 9	88 12	65 8	89 11
Education				
Upper secondary school University/higher education Other	11 64 3	14 82 4	16 53 4	22 73 5
Work situation (*n* = 96)				
Employed Not working Missing	58 20 –	74 26 –	56 16 1	77 22 1
Main diagnosis as reported by participants (*n* = 151)				
Depression/anxiety disorders Schizophrenia spectrum/psychotic disorders (including bipolar disorder) Autism spectrum/neurodevelopmental disorders Other (including personality disorders) Missing	30 19 7 13 9	38 24 9 17 12	22 26 7 10 8	30 35 9 14 11

A link to the post-intervention assessments (T2) was sent to all participants in both groups that had not actively dropped out, regardless of training time (Fig. 1). This also included registered participants with 0 minute’s training time that had not actively dropped out. The assessments also included questions about usability, confounding factors, and negative training effects post-intervention (T2) and at the 3-month follow-up (T3). Out of the 76 participants in the experiment group and the 71 participants in the WLC who received a link to the post-intervention assessments (T2), 75% (*n* = 57) and 89% (*n* = 63) respectively answered. Follow-up assessments (T3) were sent to the participants 3 months after termination of the respective test periods at which point 37 participants in the experiment group answered and 33 WLC.

Daily formal practice time was registered online through the program’s website (120 min = 1 week’s training). The total number of missing cases regarding training time was 26 for both groups, with 7 in the experiment group and 19 in the WLC. This is due to participants not registering or registering onto the program website, but not initiating their training. These cases have been included in the 0–120 min interval below. If training was initiated, but did not proceed up to the fulfillment of the first 10 minutes’ exercise, the training time was registered as 0 min (experiment group *n* = 7, WLC *n* = 16) (=pre-treatment drop-out). In the experiment group at T2, 35% (*n* = 27) hence had a training time between 0 and 120 min, 14% (*n* = 11) between 120 and 480 min, and 51% (*n* = 40) between 481 and 960 min, whereas the figures in the respective training time intervals at the 3-month follow-up were 35% (*n* = 27), 10% (*n* = 8), and 55% (*n* = 43). For the WLC, the figures were 62% (*n* = 45, including pre-treatment drop-outs) in the 0–120 min training interval, 7% (*n* = 5) in the 121–480 min interval, and 31% (*n* = 23) in the 481–960 min interval at T2, and 62% (*n* = 45), 5% (*n* = 4), and 33% (*n* = 24) in the respective training time intervals at T3.

### Procedure

The current effectiveness study was designed as a randomized controlled trial with an experimental group and a wait-list control group (WLC), with measurements at baseline, post-intervention, and at a 3-month follow-up on primary and secondary outcomes and usability. The WLC was offered the same program after termination of the experiment group’s test period.

#### Intervention

The intervention consists of a web-based mindfulness program specifically tailored for families living with MI in the sense that its contents were related to carers’ situation and associated experiences of burden and stress. The program could be accessed through a computer/tablet/smartphone with Internet access. It consists of audio/video files (960 min) accompanied by written keywords on the screen, descriptive text files, and instructions for daily mindfulness exercises, including (self) compassion exercises, a time log, and a private diary (not visible to the researchers). The recommended training was set to 2 × 10 min/day, 6 days/week for 8 consecutive weeks. It included basic mindfulness practices such as breathing exercises, body scans, mindful yoga/conscious movements, attention to experiences through the senses, and (self) compassion meditations. These exercises are similar to those that can be found in MBSR programs; however, they were kept to a maximum of 10 min/exercise in the current program to make them more easily practicable for participants with already hectic schedules (Table 2). To allow for individual flexibility, the test period was set to 10 weeks for both groups. Weekly e-mail reminders, including contact information to the research group/technical support, were sent to the participants as reminders/motivators for training.

**Table 2:** The 8-week program’s 8 steps (English translation)

Week	Contents
1 2 3 4 5 6 7 8	The breathing body Being present in the body Mindfulness in life and movement Compassion with the self-acceptance Wonderful pleasure Being whole Compassion with others To live with the possibility of choosing

### Measures

Participants were sent a link for data collection with a sociodemographic questionnaire (only at T1) and validated self-assessments scales online at baseline (T1), post-intervention (T2), and at a 3-month follow-up (T3).

#### Five Facet Mindfulness Questionnaire (FFMQ)

It consists of 39 items, rated on a 5-point Likert scale (1 = never or very rarely true, 5 = very often or always true), assessing five facets of mindfulness: Non-reactivity to inner experience (7 items), Observing (8 items), Acting with Awareness (8 items), Describing (8 items), and Non-judging of Experience (8 items). The scale has shown good internal consistency with alpha coefficients ranging from 0.75 to 0.91 (Baer et al. 2006, 2008). The Swedish version of the FFMQ has shown good psychometric properties, with results comparable to those obtained by Baer et al. (Baer et al. 2006, 2008; Lilja et al. 2011). Cronbach’s alpha for FFMQ in the current study was 0.92.

#### CarerQoL7-D

This self-rating instrument measures seven dimensions of caregiver burden using seven items with a 3-point response scale (0 = no problems to 3 = a lot of problems): fulfillment, relational dimension, mental health dimension, social dimension, financial dimension, perceived support, and physical dimension. It includes the CarerQoL-VAS, indicating the level of happiness with caregivers’ experiences and encompassing both negative and positive aspects, ranging from 0 = “completely unhappy” to 10 = “completely happy” (Brouwer et al. 2006). The scale has shown good validity in measuring informal carer effects (Brouwer et al. 2006; Hoefman et al. 2011).

#### Self-Compassion Scale-Short Form (SCS-SF)

This 12-item scale measures six components of self-compassion using six subscales with two items each: Self-Kindness, Self-Judgment, Common Humanity, Isolation, Mindfulness, and Over-Identification. Items are rated on a 5-point response scale (1 = almost never to 5 = almost always) (Raes et al. 2011). The scale has shown adequate internal consistency (Cronbach’s alpha ≥ 0.86) and a near-perfect correlation with the long-form SCS (*r* ≥ 0.97) (Raes et al. 2011). The Swedish version of the SCS was translated and back-translated by Strömberg (unpublished manuscript) and approved by Neff, the scale’s originator. It showed good reliability in a Swedish study (Wallmark et al. 2013). A short version was used in the present study, for which Cronbach’s alpha in the current study was 0.86.

#### Perceived Stress Scale (PSS)

It is a validated 14-item scale measuring the degree to which situations in life in the past month are appraised as unpredictable, uncontrollable, and overwhelming, using a 5-point response scale (0 = rarely to 4 = very often) (Cohen 1988). The scale has shown good reliability and validity. The Swedish version has demonstrated good internal consistency (0.82) and split-half reliability (0.84), and adequate construct validity (Eskin and Parr 1996). Cronbach’s alpha for PSS in the current study was 0.74.

#### Usability, confounding factors, and negative effects of training

A Swedish version of the System Usability Scale (SUS) (Brooke 1996) was used to assess the program’s usability. It is a 10-item 5-point Likert scale giving a global view of subjective assessments of usability. Possible scores range between 0 and 100 with higher scores indicating better usability. A system with a SUS value >70 can be estimated as good and >85 as excellent, although it does not guarantee high acceptability in the field (Bangor et al. 2008). Additional questions with room for free-text answers about usability, confounding factors (other sources of support, negative life events, patient’s health status), and negative effects of training were also included for separate analysis.

## Data Analyses

General linear model analyses with repeated measures and analyses of variance were performed to evaluate the intervention’s impact on primary (FFMQ) and secondary (SCS-SF, PSS) outcomes as compared to WLC. Paired-samples *t* tests were carried out to compare means on caregiver burden (CarerQoL7-D). Between-group effect sizes for primary and secondary outcomes for the experiment group and the WLC post-intervention were calculated, and within-group effect sizes for the experiment group at follow-up, using Cohen’s *d*. Effect sizes below 0.5 were considered as small, between 0.5 and 0.8 as medium, and above 0.8 as large (Cohen 2013). IBM SPSS Statistics package version 22 was used in all statistical analyses. Spearman correlation analyses were used to investigate the association between amount of exercise reported and outcome of the intervention.

A total score was calculated to obtain an overall value of system usability (SUS) (Brooke 1996). Qualitative data from the usability questionnaires (T2, T3) was analyzed with content analysis (Graneheim and Lundman 2004) and quantitative data is reported with descriptive statistics. The program was similar for both groups (experiment and WLC). Since usability data was available and can contribute to enhance the intervention, usability data from both groups was aggregated (Tables 5 and 6).

## Results

Mindfulness (FFMQ) significantly improved pre- and post-intervention in comparisons between the experiment group and the WLC group. This was the case for both the overall score and all subscale scores (*p* = 0.001) with effect sizes mainly medium or large, range 0.42–1.1. There were significant and positive improvements in the experiment group between pre-intervention and follow-up in both the overall and the subscale scores with *p* values 0.001 except for the subscale non-judging (*p* = 0.005) and within-group effect sizes were in the range 0.50–0.97 (Table 3).

**Table 3:** Outcome of pre- and post-intervention comparisons between groups regarding Mindfulness, Self-Compassion, and Perceived Stress (repeated measures ANOVA) and pre-intervention follow-up in the experimental group (paired-samples *t* test)

Outcome measure	Baseline		Post-intervention			Pre- and post-intervention	Follow-up		Pre-intervention follow-up
	Experiment group (*n* = 56)	WLC (*n* = 63)	Experiment group (*n* = 56)	WLC (*n* = 63)			Experiment group (*n* = 37)		
	M ± SD	M ± SD	M ± SD	M ± SD	*p* value	Effect size	M ± SD	*p* value	Effect size
FFQM global sum Non-reactivity Observing Acting with awareness Describing Non-judging	118.4 ± 23.1 17.6 ± 5.1 24.1 ± 6.0 23.2 ± 7.1 26.5 ± 7.5 26.9 ± 7.2	122.9 ± 18.8 19.8 ± 4.7 25.6 ± 5.7 24.1 ± 6.3 27.6 ± 5.7 25.9 ± 7.0	134.8 ± 18.5 22.2 ± 4.2 27.6 ± 5.7 26.4 ± 5.3 28.5 ± 6.6 30.0 ± 6.3	120.4 ± 20.2 19.3 ± 4.1 25.4 ± 6.2 23.4 ± 7.2 26.9 ± 6.4 25.3 ± 6.5	0.001 0.001 0.001 0.001 0.001 0.001	0.94 1.1 0.63 0.60 0.42 0.55	136.1 ± 20.4 22.3 ± 4.7 27.8 ± 5.0 27.3 ± 5.7 29.6 ± 6.7 29.1 ± 6.5	0.001 0.001 0.001 0.001 0.001 0.005	0.97 0.87 0.68 0.82 0.50 0.56
SCS-SF global sum Self-Kindness Self-Judgment Common Humanity Isolation Mindfulness Over-identification	32.7 ± 9.4 5.4 ± 1.7 6.0 ± 2.4 5.8 ± 2.0 7.1 ± 2.3 6.4 ± 2.0 7.9 ± 1.9	34.8 ± 8.1 5.4 ± 1.5 5.8 ± 1.9 6.3 ± 1.9 6.9 ± 2.1 7.3 ± 1.8 7.5 ± 2.0	38.6 ± 7.8 6.2 ± 1.6 4.7 ± 2.0 6.9 ± 1.7 6.3 ± 2.0 7.1 ± 1.6 6.6 ± 2.0	33.8 ± 8.9 5.7 ± 2.0 6.0 ± 2.2 6.1 ± 2.0 7.0 ± 2.1 6.9 ± 1.9 7.8 ± 2.0	0.001 0.048 0.001 0.001 0.003 0.002 0.001	0.80 0.30 0.70 0.68 0.42 0.61 0.80	38.4 ± 3.6 6.4 ± 1.6 7.1 ± 1.8 7.0 ± 1.7 5.6 ± 2.3 7.3 ± 1.6 5.5 ± 2.1	0.001 0.001 0.079 0.001 0.012 0.009 0.001	1.0 0.63 −0.50 0.72 0.75 0.49 1.2
PSS	33.0 ± 6.7	32.9 ± 7.1	28.8 ± 6.8	33.4 ± 7.8	0.002	0.66	28.6 ± 8.2	0.002	0.66

*p < 0.05

There were also significant improvements pre- and post-intervention regarding self-compassion in comparisons between the groups. The experiment group showed significant changes in the global SCS-SF score and in several of the SCS-SF’s subscales at the *p* = 0.001 level, except for the Self-kindness (*p* = 0.048), Isolation (*p* = 0.003), and Mindfulness (*p* = 0.002) subscales. Effect sizes were small to medium (range 0.30–0.80). Comparisons pre-intervention and follow-up showed significant within-group improvements in the experiment group in all subscales except for the Self-Judgment scale, with effect sizes in the range 0.49–1.2.

Significant decreases in favor of the experimental group were observed in perceived stress (PSS scale) in pre- and post-intervention comparisons between the groups (*p* = 0.002 and ES = 0.66). Within-group comparisons in the experimental group showed a significant improvement between pre-intervention and follow-up (*p* = 0.002 and ES = 0.66).

Regarding CarerQoL-7D, comparisons between the groups pre- and post-intervention showed significant improvements in favor of the experimental group in three out of seven dimensions, relational problems, mental health, and problems with daily activities (effect sizes ranging from 0.30 to 0.57, Table 4). Within-group comparisons pre-intervention and follow-up in the experiment group showed improvements in fulfillment (*p* = 0.01, ES = 0.46) and relational problems (*p* = 0.002, ES = 0.62). No improvements were shown in the other dimensions including the VAS scale which indicates levels of happiness with caregiving experiences.

**Table 4:** Paired-samples test and effect size for 7 dimensions of caregiver burden and CarerQoL-VAS

Caregiver burden (Carer QoL-7D)		Baseline				Post-intervention					Pre- and post-intervention	Follow-up			Pre-intervention follow-up
		Experiment group (*n* = 57)		WLC (*n* = 63)		Experiment group (*n* = 57)		WLC (*n* = 63)				Experiment group (*n* = 37)			
		Mean	SD	Mean	SD	Mean	SD	Mean	SD	*p* value	Effect size	Mean	SD	*p* value	Effect size
	Fulfillment	1.95	0.65	1.95	0.61	2.09	0.65	1.92	0.60	0.135	0.32	2.11	0.67	0.010	0.46
	Relational problems	2.25	0.64	2.24	0.76	1.89	0.65	2.19	0.72	0.019	0.57	1.84	0.73	0.002	0.62
	Mental health problems	2.20	0.67	2.15	0.62	1.98	0.61	2.24	0.67	0.009	0.30	2.14	0.48	0.499	0.13
	Problems with daily activities	1.91	0.69	1.89	0.65	1.68	0.66	1.94	0.67	0.023	0.30	1.70	0.74	0.095	0.36
	Financial problems	1.45	0.68	1.33	0.57	1.41	0.70	1.36	0.63	0.454	0.30	1.32	0.63	0.534	0.10
	Support	1.73	0.67	1.84	0.68	1.79	0.73	1.85	0.67	0.752	0.00	1.94	0.72	0.109	0.30
	Physical problems	1.79	0.78	1.85	0.72	1.70	0.60	1.79	0.68	0.845	0.00	1.70	0.64	0.169	0.17
	CarerQoL-VAS	4.32	2.13	4.33	2.53	5.38	2.20	4.73	2.70	0.158	0.25	4.92	2.37	0.219	0.28

*p < 0.05

Out of the 99 participants (56 in the experiment group and 43 in the WLC) answering the usability questionnaire SUS at T2 after testing the program, 22% reported overall SUS scores with values <70, 29% reported scores ≥70 (good), and 49% had scores ≥85 (excellent). Motivators and barriers to use related to technical aspects (e.g., flexibility and ease of use vs. navigation difficulties), program contents (e.g., guiding voice vs. lack of exercise variation), and the participants’ life situation (e.g., hectic schedule) were described in the free-text answers, where effects of training were described as motivating (Tables 5 and 6) and e-mail reminders were experienced as positive for training by almost two thirds of the participants. Some negative effects were reported by a minority of participants, out of which experiencing the training as another stressful demand was the most common. Negative life events during the experiment group’s test period were reported by over a third of participants in both groups and roughly as many reported having additional sources of support. A majority did or would recommend the program to others and as many considered pursuing their training, which they also reported doing to different degrees after the test period’s termination.

**Table 5:** Aspects of usability and value as extracted from the free-text answers at T2/T3 (experiment and control group)

Technical advantages and motivation to use	Related to technology: Easy access, independence and flexibility of use (place, time, technical platform, independent use) Ease of use Related to contents: Speaker’s voice as guide—agreeable and easy to follow Effects as motivator: Noticing positive effects of training (e.g., relaxation, calmness, better sleep, conscious awareness, self-insight, reflection on feelings/thoughts/behavior, being here and now, tool to cope with worry/stress/difficult situations, non-judgment towards self and others, more peaceful relationships, feeling whole, breaking automatic behavior)
Disadvantages and barriers to use	Related to technology: Technical difficulties (e.g., log-in procedure, insecurity regarding registration of training time online), defect computer Navigation difficulties (crowded website, too many clicks) Unstable/unavailable Internet connection (e.g., when traveling/commuting) Related to contents: Lack of variation (exercises, instructions, speaker’s voice) Too much talking (wish for more silent periods) Certain contents provoking/difficult to relate to/too abstract/not experienced as relevant for one’s situation Training induces a tendency to feel like a victim by accepting the situation as it is (as suggested in mindfulness training) Related to own situation: Lack of time and/or quiet space to do the training Life events (e.g., own and/or family health issues, fatalities) Difficulties with discipline, time, and concentration
Other feedback and suggestions	Related to technology: Internet-independent application Possibility to browse through the exercises and to create own playlists Easier navigation (on website/smartphones) Clearer instructions (e.g., regarding registration of training time, written instructions) More varied exercises, although the repetitive format easy to grasp Fewer steps or longer test period (to counteract the perception of training as another stressful demand) Reminders (e.g., push notices in the mobile phone) Related to contents: Partly “foreign” or abstract language (e.g., compassion training, certain figures of speech) Clearer focus on carers’ situation with more tangible advice
Negative effects of training	Experiencing the training as another stressful demand, not training induces guilt feelings One negative experience from too intense training when trying to catch up/solved it by talking to friends Stressful experience to focus on the body Stirs up feelings Insight into own behavior both positive (aha experience) and negative (realization of “bad” choices and priorities) Early awakening Gets cold during meditation when sitting/lying still Commercial feel (too much advertisement but otherwise good)
Confounding factors	Other sources of support: Own support from health professional (e.g., psychologist, therapist, physician) Caregiver support Emotional/practical support from friends/family/colleagues Work-related support (e.g., partial return to work) Other sources of support (e.g., books, yoga, holiday, etc.) Professional support for the patient (e.g., medication, assisted living) Negative life events: Ill health, conflicts or fatalities in family (very common) Deterioration in patient’s health (mental, physical) Own health deterioration (physical, mental) Work-related problems Economic deterioration due to own or patient’s illness Other (moving, not being heard in health care system)

**Table 6:** Usability aspects and potential confounding factors as experienced during the test period and at the 3-month follow-up (free-text answers and descriptive statistics, T2/T3)

Assessments at T2—after the experiment group’s test period	Experiment *n* = 56	WLC *n* = 63
Health fluctuations in the patient during the test period		
Improved Unchanged Deteriorated Fluctuated (better and worse) Don’t know	30% 32% 4% 20% 14%	11% 27% 16% 38% 8%
Support from other sources during the test period with potential positive effects		
Yes No Don’t know	30% 68% 2%	36% 62% 2%
Life events during the test period with potential negative effects		
Yes No Don’t know	34% 59% 7%	38% 56% 6%
Assessments at T2—after the experiment group’s and the WLC’s respective test periods	Experiment *n* = 56	WLC *n* = 43
Negative effects of training		
Yes No Don’t know	18% 77% 5%	16% 79% 5%
e-mail reminders positive for training		
Yes No Neither nor Don’t know	57% 7% 32% 4%	58% 10% 30% 2%
Potentially pursue training with a similar program (T2)		
Yes Maybe No Don’t know	75% 14% 9% 2%	67% 21% 7% 5%
Would/has recommend(ed) the program to others		
Yes Maybe No Don’t know	73% 20% 2% 5%	72% 9% 9% 9%
Assessments at T3—3 months after termination of the respective test periods	Experiment *n* = 37	WLC *n* = 33
Continued training after test period		
Yes, 4–7 days/week Yes, 2–3 days/week Yes, 1–7 days/month No, hardly ever	22% 8% 46% 24%	12% 15% 46% 27%
Experienced continued training after test period as valuable		
Yes, very Yes, partly No, hardly or not at all Don’t know Missing	30% 46% 8% 5% 11%	27% 34% 15% 18% 6%
Potentially pursue training with a similar program (T3)		
Yes Maybe No Don’t know	78% 16% 3% 3%	64% 27% 3% 6%

### Amount of exercise

We examined whether there was a relationship between the amount of exercises performed during the intervention and changes in mindfulness through a correlational analysis. It showed a significant moderate association of 0.53 pre- and post-intervention and 0.44 between pre-intervention and follow-up, indicating that amount of exercise accounted for around 28 and 19.4%, respectively, of the variation in changes in mindfulness. Considering the substantial number of participants dropping out from the investigation between assessments at termination of intervention and 3-month follow-up, we analyzed whether dropouts differed from remainders with regard to outcome pre- and post-intervention in the primary outcome measure mindfulness. This analysis showed no differences between the groups regarding changes in mindfulness, which indicates that those participating in the follow-up were representative for the sample participating in the intervention.

## Discussion

Similarly to the earlier feasibility study, significant improvements were found in the primary outcome mindfulness, with a large effect size in the mindfulness global sum and mainly medium to large effect sizes in all subscales both post-intervention and at follow-up. Significant improvements were also found in the secondary outcomes self-compassion and perceived stress, with mostly medium-sized effects. The current study showed that training time was moderately associated with changes in the primary outcome mindfulness, and that there were no differences between drop-outs and those remaining in the intervention regarding mindfulness outcomes, speaking for representativeness of the group. Recent studies show that even shorter interventions have beneficial health outcomes (Boettcher et al. 2014; Carmody et al. 2009; Krusche et al. 2012; Stjernswärd and Hansson 2016a; Zeidan et al. 2010). Neurobiological hypotheses suggest that sustained training leads to neuroplastic structure and function changes in the brain, where mindfulness states as cultivated through training may result in increased mindfulness as trait (Garland et al. 2010; Kiken et al. 2015). An interesting question is whether MBI are more beneficial for some individuals than others. Individual trajectories of change in state mindfulness have been found to predict changes in trait mindfulness and distress pre- and post-intervention (Kiken et al. 2015). Although nothing can be said about this in the current study, it is an interesting line of research and further studies may shed light onto the meaning of individual differences for outcomes in mindfulness training.

Surprisingly, caregiver burden as measured by the CarerQoL7-D in most aspects did not show significant improvements, with a few exceptions including the relational dimension. These findings challenge our hypothesis that mindfulness training can reduce caregiver burden and previous research on MBI for caregivers (Epstein-Lubow et al. 2011; Stjernswärd and Hansson 2016a). A mindful attitude has previously been found to be negatively correlated with caregiver burden, depression, and anxiety and positively correlated with quality of life (Pagnini et al. 2015). A possible explanation for the current findings, besides lack of effect of the intervention, may be that the instrument is “rough-grained” or that the outcome is suboptimal in the present context. Maybe alternative instruments measuring objective and subjective dimensions of caregiver burden could be tested in future studies. The improvements in mindfulness, self-compassion, and perceived stress may nevertheless contribute to enhance caregivers’ quality of life and ability to cope with the daily stresses engendered by a life with MI, somehow decreasing their experiences of burden.

Noteworthy in the present findings is that the self-compassion global sum and the over-identification subscale went from medium-sized effects post-intervention to large at follow-up, which may be positive for caregivers. An intense self-focus following the confrontation of one’s own limitations can lead to tunnel vision, over-identification, and being carried away with negative thoughts and feelings about the self (Neff and Vonk 2009). Self-directed compassion generates a desire to alleviate one’s own suffering, to heal oneself with kindness, to recognize one’s shared humanity, and to be mindful when considering negative aspects of oneself (Neff 2003; Neff and Vonk 2009; Thompson and Waltz 2008). Mindfulness and compassion can help tame the inner critic (Stjernswärd and Hansson 2016b), reduce guilt and stress, and improve interpersonal relationships (Hofmann et al. 2010; Jazaieri et al. 2014; Stjernswärd and Hansson 2016b; Yadavaia et al. 2014). A mindful attitude may be protective against caregiver burden in several ways, for instance by not remaining trapped and defining oneself as a caregiver only and thereby limiting one’s identity (Pagnini et al. 2015). To regain an identity separate of that of a caregiver, caregivers need to allow themselves to re-evaluate priorities and gain more balance between their relationships and other domains of life, such as occupational or leisure activities (Priestley and McPherson 2016; Stjernswärd and Östman 2008).

Burnout is common among caregivers (Onwumere et al. 2015), who also report difficulties in balancing relationships and activities in daily life (Priestley and McPherson 2016; Stjernswärd and Östman 2008). Caregivers struggle with strong emotions, including worry, (self) stigma, and blame (Eaton et al. 2016; Stjernswärd and Östman 2008; Stjernswärd and Hansson 2016b). Low levels of self-compassion have been associated with self-criticism, guilt, rumination, and worry (Raes 2010), and caregivers with low levels of self-compassion that tend to continually subordinate own needs risk compassion fatigue and burnout (Ringenbach 2009). Interventions that raise self-compassion can raise positive affect and reduce negative affect, shame, and emotional exhaustion (Leary et al. 2007; Neff et al. 2007b; Neff and Vonk 2009). Self-compassion supports an increased acceptance of both pleasant and unpleasant experiences and a non-judgmental acceptance of present moment experiences. It has been associated with greater likelihood to compromise in conflict situations and lesser likelihood to subordinate own needs, hence acknowledging both own and others’ needs (Yarnell and Neff 2013), which is central to healthy interpersonal relationships (Grotevant and Cooper 1986). Self-compassion has been associated with healthier and more sustainable interactions (Crocker and Canevello 2008), greater authenticity, relational well-being (Neely et al. 2009; Yarnell and Neff 2013), self-reported life satisfaction (Neff et al. 2007b; Neff and Vonk 2009), less emotional turmoil (Yarnell and Neff 2013), and has been negatively associated with anxiety and depression (Neff et al. 2007a; Raes 2010; Ying 2009). It can be an antidote against unproductive repetitive thinking such as rumination and anxious worrying, and act as a buffer against anxiety and depression (Raes 2010).

Most participants in the current sample were middle-aged, well-educated women and were a parent or partner—or an adult child in the WLC—to the patient. It goes in line with previous studies on web-based support (Stjernswärd 2009; Stjernswärd and Hansson 2013; Stjernsward and Hansson 2014) and MBI for caregivers (Stjernswärd and Hansson 2016a; Whitebird et al. 2011) and other populations (Fish et al. 2016). As mothers (Finley 1989), spouses, and adult children often have a major role in caregiving (Brody 1985), this is not surprising. Women are also more prone to seeking help online than men (Ybarra and Suman 2006). MBI have nevertheless been found to be effective for a variety of populations and may hence benefit a diversity of caregivers. Reaching out to caregivers in need is essential. So is finding ways to attract them and sustain engagement and motivation to participate in for instance MBI. Group-based interventions include challenges such as time commitment, willingness to engage, and travel (Whitebird et al. 2011). Web-based interventions such as the current MBI may help overcome such challenges as seen in the previous feasibility and usability studies, although they still require discipline and time commitment. This may be experienced as a stressful factor per se (Stjernswärd and Hansson 2016a, b). Ease and convenience of use and the effects of training were strong motivators for use (Stjernswärd and Hansson 2016b), also in the current study. The majority of participants rated the program’s usability as good-excellent and experienced benefits from their continued training. Nevertheless, there is room for improvement, both related to the contents and technology. Some negative effects of training were reported, e.g., stress or distressing feelings in connection with the training. Supplementing the current intervention with virtual classrooms in which participants can discuss their experiences with an instructor and/or peers may be a useful complement through which potential negative effects can be addressed, although further studies are needed to explore the value and effects of such blended interventions.

Pre-emptive policies to identify caregivers at early stages and the inclusion of assessments of caregiving activities/roles to tailor relevant and suitable interventions are called for (Smith et al. 2014b). Raising awareness about interactions with the patient, distancing oneself from problems, and seeing them from a different perspective may empower families dealing with crises such as the onset of an illness (Gavois et al. 2006). This can be achieved in interaction with a mental health professional (Gavois et al. 2006), but possibly also through MBIs such as the current one, where participants report a sense of increased awareness, perspective, and freedom of choice subsequent to their training (Stjernswärd and Hansson 2016a, b). Carers’ belief about the negative consequences of an illness for themselves have been identified as predictors of emotional exhaustion, and low personal accomplishment has been associated with a carer’s less optimistic beliefs about the illness timeline and fewer reports of adaptive coping (Onwumere et al. 2015). Tailored interventions to prevent burnout may thus be wanted, including strategies for more balanced appraisal of illness, problem-focused coping, and therapeutic optimism (Onwumere et al. 2015). MBIs such as the current one can help break automatic reactions and ruminations and facilitate more creative and strategic thinking (Stjernswärd and Hansson 2016b), which may be useful in filling the existing gap in addressing caregivers’ needs of support.

### Limitations

Dropout rates from internet-based treatments for psychological disorders indicate a 2–83% range and an average of 35% (median 24%) (Melville et al. 2010). Drop-out rates in the current study—including both non-usage and dropout attrition (Eysenbach 2005)—were 27% (*n* = 21) and 26% (*n* = 20) at T2 and T3, respectively, for the experiment group (Fig. 1). Dropout rates in earlier MBI online have shown a wide range (7.7–52.3%) (Fish et al. 2016). Recruiting enough participants to two study arms without losing interested ones while waiting for enough participants to start the study can be a challenge (Whitebird et al. 2011). Although enough participants enrolled, the current study most probably lost participants in the WLC due to time passing and the WLC’s test period’s occurrence during winter holidays (December). Interventions with an individualized start may help overcome such issues. Retaining participants in online interventions can be challenging, as also seen in the current study, even though features such as technical support, the possibility to contact the research group, and e-mail reminders were incorporated to help sustain motivation and prevent attrition. Studies encompassing comparisons of face-to-face versus web-based MBI may help shed light onto the shortcomings and benefits of the different versions, as also suggested by Tunney et al. (2016), also in light of post-intervention assessments.

It is difficult to know whether any confounding factors may have affected the results. An attempt at controlling this was made by asking participants about other sources of support, negative life events, and fluctuations in the patient’s health status occurring during the test period. Roughly a third of the participants reported valuable support from other sources and life events that affected them negatively during the test period; still, positive improvements from the intervention were found, speaking for its value. After considering the option to include measures of participants’ own mental health (e.g., anxiety, depression) in the current study, the authors opted not to so as not to burden the participants with too many questions. However, such measures may be relevant to explore further in the context of MBIs for caregivers, not the least in light of the burgeoning literature in the area. There may be insecurity in self-reported diagnoses, with a majority of depression/anxiety and schizophrenia spectrum/psychotic disorders, possibly representing common caregiver target groups with needs of support. Nevertheless, the intervention seems acceptable for caregivers of persons with a diversity of mental health conditions, representing an asset.
